# Effects of Anxious Depression on Antidepressant Treatment Response

**DOI:** 10.3390/ijms242417128

**Published:** 2023-12-05

**Authors:** Chantal Hampf, Maike Scherf-Clavel, Carolin Weiß, Catherina Klüpfel, Saskia Stonawski, Leif Hommers, Katharina Lichter, Angelika Erhardt-Lehmann, Stefan Unterecker, Katharina Domschke, Sarah Kittel-Schneider, Andreas Menke, Jürgen Deckert, Heike Weber

**Affiliations:** 1 Department of Psychiatry, Psychosomatics and Psychotherapy, Center of Mental Health, University Hospital of Würzburg, Margarete-Höppel-Platz 1, 97080 Würzburg, Germany; hampf_c@ukw.de (C.H.); deckert_j@ukw.de (J.D.); 2 Interdisciplinary Center for Clinical Research, University Hospital of Würzburg, 97080 Würzburg, Germany; 3 Comprehensive Heart Failure Center (CHFC), University Hospital of Würzburg, 97080 Würzburg, Germany; 4 Department of Psychiatry, Max Planck Institute of Psychiatry, 80804 Munich, Germany; 5 Department of Psychiatry and Psychotherapy, Medical Center–University of Freiburg, Faculty of Medicine, University of Freiburg, 79106 Freiburg, Germany; 6 Department of Psychiatry and Neurobehavioural Science, University College Cork, T12 YN60 Cork, Ireland; 7 Department of Psychosomatic Medicine and Psychotherapy, Medical Park Chiemseeblick, 83233 Bernau, Germany; 8 Department of Psychiatry and Psychotherapy, University Hospital, Ludwig Maximilian University of Munich, 80539 Munich, Germany

**Keywords:** pharmacotherapy, depressive disorder, anxious depression, anxiety, therapy response

## Abstract

Anxious depression represents a subtype of major depressive disorder and is associated with increased suicidality, severity, chronicity and lower treatment response. Only a few studies have investigated the differences between anxious depressed (aMDD) and non-anxious depressed (naMDD) patients regarding treatment dosage, serum-concentration and drug-specific treatment response. In our naturalistic and prospective study, we investigated whether the effectiveness of therapy including antidepressants (SSRI, SNRI, NaSSA, tricyclics and combinations) in aMDD patients differs significantly from that in naMDD patients. In a sample of 346 patients, we calculated the anxiety somatization factor (ASF) and defined treatment response as a reduction (≥50%) in the Hamilton Depression Rating Scale (HDRS)-21 score after 7 weeks of pharmacological treatment. We did not observe an association between therapy response and the baseline ASF-scores, or differences in therapy outcomes between aMDD and naMDD patients. However, non-responders had higher ASF-scores, and at week 7 aMDD patients displayed a worse therapy outcome than naMDD patients. In subgroup analyses for different antidepressant drugs, venlafaxine-treated aMDD patients showed a significantly worse outcome at week 7. Future prospective, randomized-controlled studies should address the question of a worse therapy outcome in aMDD patients for different psychopharmaceuticals individually.

## 1. Introduction

Major depressive disorder (MDD) is a severely disabling mood disorder which is one of the most common mental disorders in Europe (lifetime prevalence; 12.8%) and the USA (lifetime prevalence; 16.2%) [1]. Managing MDD includes antidepressant psychopharmacotherapy and psychotherapy as first-line treatments. Although antidepressant drugs are effective treatment options, 30–50% of MDD patients fail to respond adequately to several pharmacotherapy approaches [2]. Moreover, even if remission is achieved, there is a 35–70% risk of relapse [3]. MDD has a very heterogeneous clinical presentation, which further complicates identifying an effective treatment. Treatment remission rates vary depending on the clinical characteristics of the patient, which indicates the existence of MDD subgroups that respond particularly well or poorly to distinct treatment options [4].

MDD is a highly complex disorder and to date, only few pathomechanisms have been identified. At the neurotransmitter level, the connection between serotonin deficiency and the development of depression was discussed as early as the 1960s, although this concept has recently been systematically reviewed and called into question [5]. Abnormal levels of dopamine and norepinephrine, neurotransmitters crucial for motivation and reward, have been observed in depressed rodents and have therefore been implicated in MDD pathogenesis [6].

A dysregulation of the hypothalamic-pituitary-adrenal (HPA) axis, which is involved in stress regulation, has also been proposed as an underlying mechanism. HPA dysregulation leads to an increase in corticotrophin-releasing hormone, adrenocorticotropic hormone, and cortisol or corticosterone [6]. The increased cortisol levels can trigger dysfunction in areas of the brain responsible for mood regulation, such as the prefrontal cortex [6]. Additionally, an overproduction of proinflammatory cytokines caused by the dysregulation of the HPA axis has been demonstrated, which is thought to contribute to the development of depression through the loss of neurogenesis in the hippocampus [6]. Lastly, neuroimaging studies have revealed structural and functional changes in the prefrontal cortex in MDD patients [7]. Consequently, brain stimulation techniques are now used to stimulate the dorsolateral prefrontal cortex in depressed patients and thus alleviate symptoms through mood modulation [7].

One of the most challenging subtypes of MDD is anxious depression with a prevalence of 45–55% [8,9]. Anxious depression can be defined dimensionally and categorically by separately analysing questions on anxiety in depression questionnaires such as the HDRS. This common subtype is associated with increased suicidality, severity, and chronicity [8,10,11,12,13]. Additionally, studies have shown that depressed patients with high anxiety levels often show a lower treatment response [9,14,15,16,17]. The reasons underlying these clinical differences in anxious depression are not clear. However, one hypothesis involves the neurovisceral integration model, which is used to elucidate anxiety at a neuronal level [18]. This model assumes that high-level cognitive structures, such as the prefrontal cortex, influence the amygdala, the insula and the hippocampus [18]. These structures then are thought to cause neurovisceral reactions via projections that influence cardiac behaviour [18]. These additional effects are postulated to contribute to the more severe and chronic course of anxious depression.

Studies investigating pharmacotherapy outcomes in anxious depressed (aMDD) patients compared to non-anxious depressed (naMDD) patients are rare, especially those with drug-specific treatment response, and have shown mixed results [9,15,19]. As anxious depression is associated with additional pathomechanisms and a high level of distress in patients, it is essential to obtain a further understanding of these subtypes and potential effective treatments. In the present study, we focused on therapy outcomes in aMDD patients to improve therapy options and response for aMDD patients. Firstly, we aimed to determine whether the success of antidepressant treatment differs significantly in aMDD from that in naMDD patients at baseline, but also in patients with lasting elevated anxiety levels who could still be classified as anxious depressed after therapy. Secondly, we were interested whether there are differences in drug-doses applied or serum-concentrations observed between the aMDD and naMDD phenotype at the start and end of therapy. Lastly, we examined drug-specific differences in treatment response depending on the anxious versus non-anxious phenotype at both time points.

## 2. Results

To investigate the effects of high-grade anxiety symptoms in depressive patients on the success of antidepressant treatment we analysed 346 depressive patients (mean age 45.58 ± 15.31 years; 57.80% female), treated with different psychopharmacological medication (Supplementary Table S2). We divided the patients into aMDD and naMDD at baseline and after seven weeks of therapy and looked at differences in treatment response between aMDD and naMDD at both time points. To determine the differential response to specific psychopharmacological medication, based on the respective drug doses or the resulting serum concentrations, sub-analyses were performed on individual drugs. Due to power reasons, sub-analyses were restricted to four antidepressant drugs whose doses and serum concentrations were available in at least 30 participants, namely amitriptyline (N_dose_ = 84; N_concentration_ = 71), mirtazapine (N_dose_ = 98; N_concentration_ = 72), sertraline (N_dose_ = 35; N_concentration_ = 32) and venlafaxine (N_dose_ = 166; N_concentration_ = 152). In the one-tailed Wilcoxon signed-rank test, to detect differences in treatment response, the sample reached a power of 85% and 78% for amitriptyline (dose and concentration, respectively), 89% and 79% for mirtazapine, 52% and 50% for sertraline and 98% and 97% for venlafaxine-treated patients with an effect size of 0.3. Detailed demographics of the total sample are summarized in Table 1 and drug-specific demographics are given in Supplementary Table S1.

### 2.1. Treatment Response in Anxious Depression

To investigate whether the efficacy of pharmacotherapy is influenced by the degree of anxious symptomatology in depressed patients, we first examined how the HDRS ASF-score effects treatment response after pharmacotherapy. We used the ASF-scores at the study baseline and also analysed the ASF-scores at week 7 to investigate patients who remained anxious depressed after therapy.

Treatment response did not differ between aMDD and naMDD patients according to baseline ASF-scores, nor to categorical classification in aMDD vs. naMDD patients at baseline.

**Figure 1:**
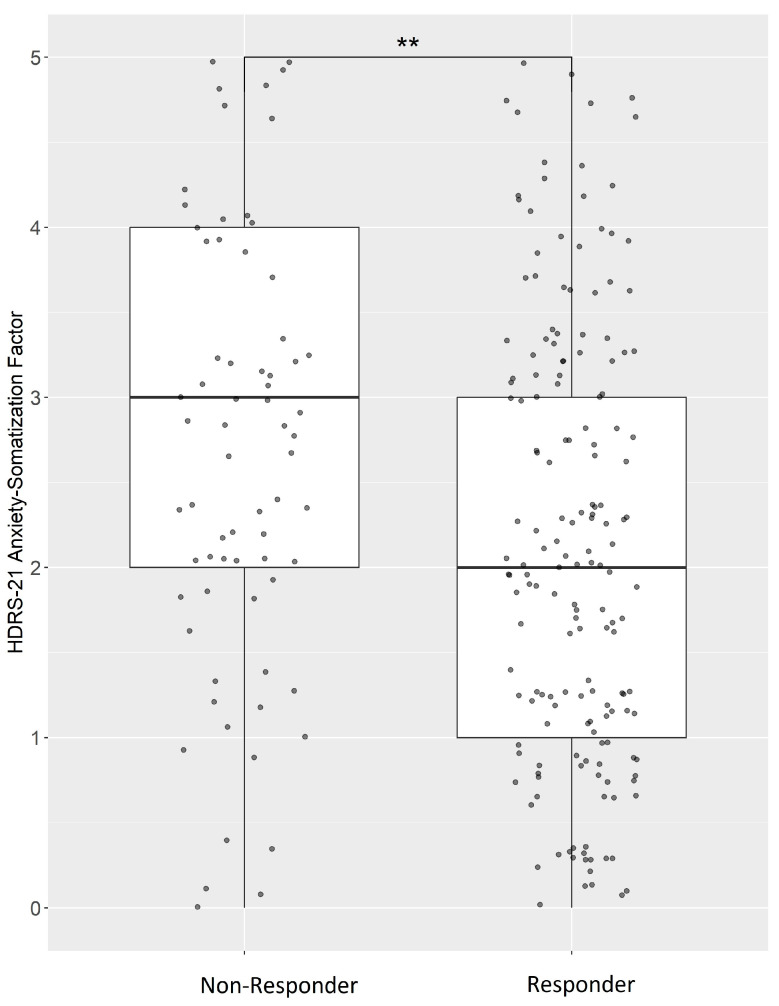
Week 7- Non-responders displayed higher anxiety-somatization factor scores. A more detailed overview of the administered psychiatric drugs is summarized in Supplementary Table S2. ** significant after Bonferroni correction.

However, when evaluating the ASF-scores after 7 weeks of therapy, non-responders had significantly higher ASF-scores compared to responders (beta = −0.34; P = 1.68 × 10^−9^; P_corrected_ = 4.37 × 10^−8^; Figure 1). After performing a correction for comedications, the results remained similar (beta = −0.32; P = 4.67 × 10^−8^; P_adjusted_ = 1.21 × 10^−6^).

**Figure 2:**
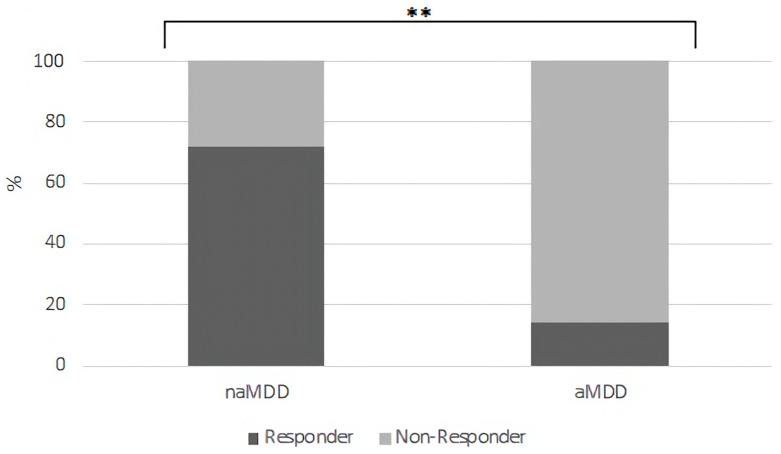
Week 7- aMDD patients displayed a worse therapy response than naMDD patients. ** significant after Bonferroni correction. N_aMDD_ = 31; N_naMDD_ = 304.

In addition, after categorizing the patients into aMDD vs. naMDD subgroups according to ASF-scores at week 7, we found that patients who show symptoms of anxious depression are significantly more likely to show non-response after 7 weeks of therapy, as indicated by the HDRS-21 sum score (beta = −2.96; P = 2.17 × 10^−7^; P_adjusted_ = 5.64 × 10^−6^; Figure 2). Therefore, patients with aMDD in week 7 showed a significantly worse therapy response than naMDD patients. The results again remained similar after correction for comedications (beta = −2.96; P = 6.78 × 10^−7^; P_adjusted_ = 1.76 × 10^−5^).

### 2.2. Drug Doses and Serum Concentrations in Anxious Depression

We next determined whether depressed patients with higher anxiety levels received higher drug doses or had elevated serum concentrations compared to patients with lower anxious symptomatology according to ASF-score.

However, neither drug doses nor serum concentrations of the four analysed antidepressant drugs (amitriptyline, mirtazapine, sertraline, venlafaxine) showed a significant association with the dimensional ASF-score, nor did they differ between the categorically aMDD and naMDD phenotype (*p*_all_ ≥ 0.05) at baseline or week 7 of therapy.

### 2.3. Drug-Specific Treatment Response in Anxious Depression

We next examined treatment response to different drugs depending on the anxiety level of depressed patients.

Drug sub-analyses revealed no differences in treatment response for either the ASF-scores or the categorical aMDD and naMDD phenotype at baseline.

**Figure 3:**
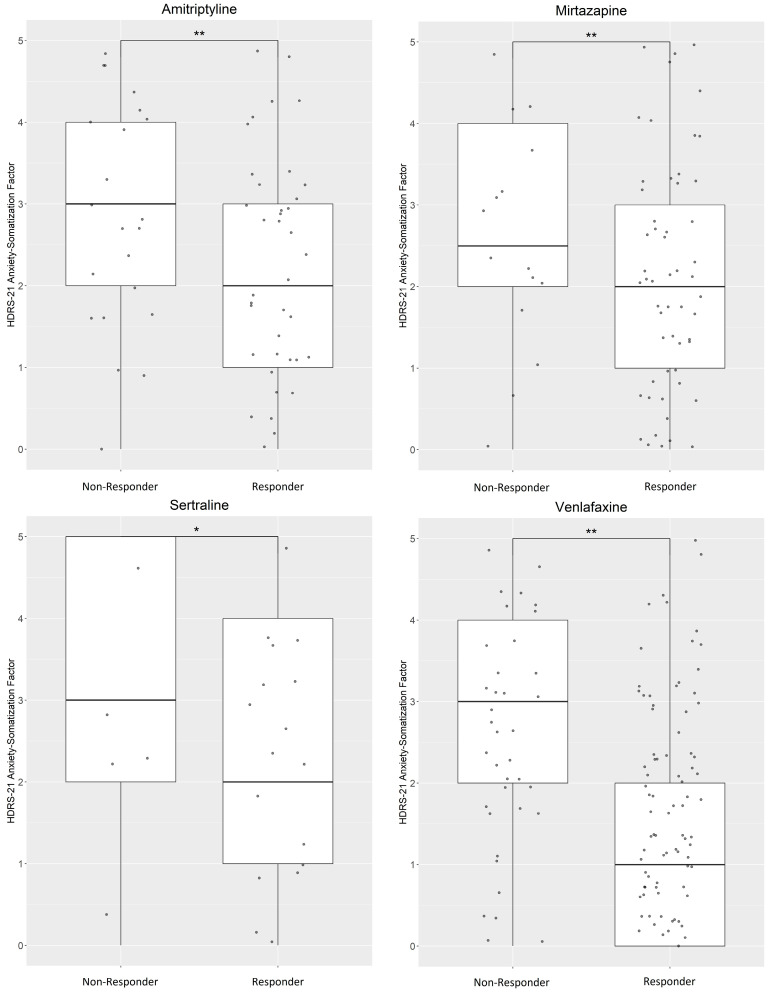
Week 7- anxiety-somatization factor scores were higher in non-responders for all medication-groups. A more detailed overview of the administered psychiatric drugs is summarized in Supplementary Table S2. * significant association (*p* < 0.05); ** significant after Bonferroni correction.

However, with regard to anxious symptomatology at week 7, we found that non-responders had higher ASF-scores in all four medication sub-samples at a nominal level; amitriptyline (N = 84; beta = −0.35; P = 1.59 × 10^−3^), mirtazapine (N = 98; beta = −0.45; P = 1.02 × 10^−4^), sertraline (N = 35; beta = −0.61; P = 0.012), venlafaxine (N = 166; beta = −0.38; P = 3.18 × 10^−6^). After correction for multiple testing, only the results of patient groups treated with amitritpyline (P_adjusted_ = 0.041), mirtazapine (P_adjusted_ = 2.65 × 10^−3^) and venlafaxine (P_adjusted_ = 8.27 × 10^−5^) remained significant (Figure 3). After performing an additional correction for the administered comedications, significance was only found in the groups treated with mirtazapine (P_adjusted_ = 4.58 × 10^−3^) and venlafaxine (P_adjusted_ = 2.07 × 10^−4^).

**Figure 4:**
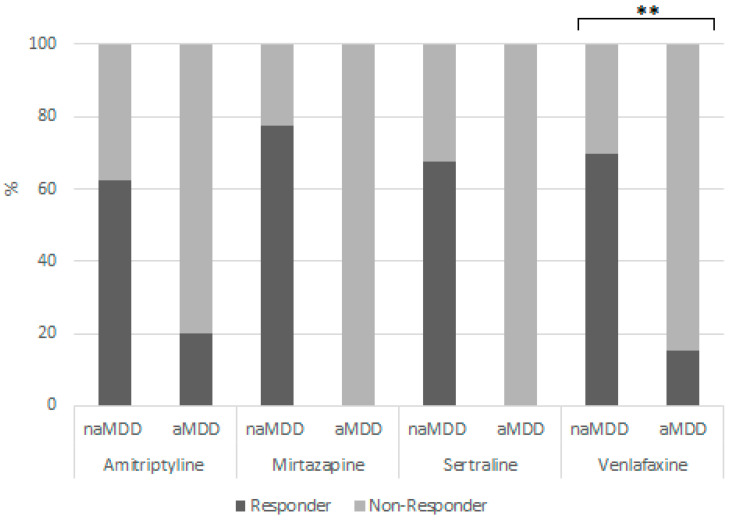
Week 7- Percentage of responders and non-responders in the naMDD and aMDD group. ** significant after Bonferroni correction.

At week 7, a significantly worse treatment response for venlafaxine-treated aMDD patients was observed (N = 166; beta = −2.80; P = 5.13 × 10^−4^; P_adjusted_ = 0.013) (Figure 4). After correction for comedications, the results remained significant (P_adjusted_ = 7.51 × 10^−3^).

In summary, non-responders did not have higher baseline ASF-scores and after categorizing the sample into aMDD and naMDD patients no differences in pharmacotherapy outcome could be observed. The results at week 7 showed that therapy non-responders had higher ASF-scores and aMDD patients displayed a worse therapy response compared to naMDD patients. We did not observe an association between drug-doses or serum-concentrations and the ASF-score, or differences between aMDD and naMDD patients. Sub-analyses of the different drug treatments showed no differences in ASF-scores, nor differences in treatment response after categorization into aMDD and naMDD patients. However, sub-analyses at week 7 showed that non-responders to all drugs had higher ASF-scores with the exception of the sertraline group. After categorization, only venlafaxine revealed an association with a worse therapy outcome in aMDD patients. The mentioned results are summarized in Table 2.

## 3. Discussion

MDD is a highly heterogeneous condition in which patients’ response to specific therapies depend on the underlying clinical phenotype [4]. Fifty (50)% of all MDD patients suffer from a comorbid anxiety disorder and this mixed phenotype is not only a common but also highly distressed subgroup [20]. Therefore, in our study we focused on pharmacotherapeutic effectiveness in high-level anxiety phenotypes. The relevance of anxiety in relation to depression has been a topic of interest for researchers since the 1960s and 1970s, when studies began exploring the heterogeneity of depression [21]. An anxiety factor or anxiety cluster was regularly identified [22,23]. The relevance of treatment was mainly related to psychiatric medications commonly used during that time, such as monoaminooxidase inhibitors or tricyclic antidepressants. In 1959, researchers observed that iproniazid could be beneficial for patients with elevated anxiety levels [24]. Two other studies from that era compared amitriptyline and phenalzine, with phenalzine showing slightly greater effectiveness for aMDD patients [25,26]. Treatment options were limited at the time, leading to the development of a broader range of medications over the next years. Consequently, numerous studies have now been conducted to examine the treatment response of aMDD vs. naMDD to different psychiatric drugs. However, these studies showed mixed results. Some studies observed that an anxious phenotype leads to a worse treatment response [9,14,15,16,17] whereas a study by Fava et al. [27] did not find significant differences. Among 24 studies on tricyclic antidepressants, one third demonstrated low efficacy in anxious depression and two thirds showed no difference between aMDD and naMDD [19]. In a pooled analysis of five venlafaxine-fluoxetine comparative studies with a sample size of 1454 patients, no differences in response rates were found between patients with moderate and high levels of anxiety [15]. However, remission rates were lower in aMDD. Three large reviews or meta-analyses that used the HDRS anxiety factor score to categorize aMDD and naMDD patients found no difference in outcome [19]. For instance, an analysis of 19 double-blind, randomized studies with fluoxetine in 3183 patients revealed no difference in the mean response rate of aMDD vs. naMDD [28]. Furthermore, in a pooled analysis of 11 placebo-controlled trials, no difference in treatment response to duloxetine was observed between aMDD and naMDD [29]. In a meta-analysis of ten double-blind, randomized trials with 2122 patients, selective serotonin reuptake inhibitors were compared with bupropion, with highly anxious and less anxious patients showing similar response rates (62.3% and 63.3%, respectively) [19,30]. In a randomized, double-blind study with sertraline and imipramine, no difference was observed between anxious and non-anxious phenotypes in the overall treatment response. However, the response of aMDD patients to the drugs appeared to be delayed [31]. According to Gaspersz et al. [32], studies that have investigated the effectiveness of pharmacological treatments in depressed patients with high levels of anxiety did not show clear results so far due to the lack of randomized-controlled studies, and the use of different definitions of anxious depression. The different definitions of anxious depression in particular seem to be a problem in the comparability of the results. For example, anxious depression has previously been defined as either a single anxiety disorder, any anxiety disorder, different anxiety disorders, scores on anxiety scales or scores on an anxiety factor of a depression scale [21].

To our knowledge, ours is one of very few studies that investigated dosage, serum-concentration and drug-specific treatment response in parallel regarding the anxiety subphenotype of MDD. Additionally, we used the ASF-score to categorize aMDD and naMDD patients dimensionally as well as categorically. This allowed us to compare our results with both studies that have used the ASF-score dimensionally or categorically and thus provide more clarity on the inconsistent results observed between previous studies regarding treatment outcome in aMDD and naMDD patients. Furthermore, to the best of our knowledge, our study is the only one that specifically analysed the remaining subgroup of patients who continued to experience anxious depression after seven weeks of therapy.

In our study, we did not observe that non-responders had a higher baseline ASF-score. Additionally, after stratifying the sample into aMDD and naMDD patients, we did not find differences in pharmacotherapy outcome. One possible explanation may be that in our study the drug-doses and serum concentrations were adjusted to higher dosages needed to successfully treat the aMDD subtype. This might be due to regular therapeutic drug monitoring as implicated in the GEParD protocols and might differ in our naturalistic treated sample from other cohorts. However, regarding differences for drug-doses and serum-concentrations, we also did not find any association between any of the four investigated drugs (amitriptyline, mirtazapine, sertraline and venlafaxine) and the dimensional ASF-score, or differences between the aMDD and naMDD phenotype in terms of therapy response.

After seven weeks of therapy, there was still a core group of 31 patients who could be categorized as aMDD patients. Analyses at week 7 showed that therapy non-responders had higher ASF-scores, accompanied by a significantly higher proportion of aMDD patients responding worse to pharmacotherapy compared to naMDD patients after categorization. Sub-analyses of the psychopharmacological medication showed no differences in ASF-scores, or in treatment response of aMDD vs. naMDD patients. In non-responders after therapy, sub-analyses of all antidepressant drugs showed higher ASF-scores, except for the smallest subgroup (sertraline). After categorization, only venlafaxine remained significantly associated with a worse therapy outcome in aMDD patients.

A potential explanation for the differing results in our naturalistic study versus other studies (e.g., STAR*D [33]) is that additional antidepressant drugs were used, in particular tricyclics, and combinations. The administration of tricyclics and combinations of SSRIs and SNRIs with mirtazapine and/or quetiapine in these severely depressed patients may have reduced differential outcomes for aMDD and naMDD. Non-significant results could also be related to the issue of antidepressants having a delayed onset of efficacy. In addition, some patients also received adjuvant treatments such as psychotherapy. These additional interventions can influence treatment response and may have contributed to the lack of significant differences observed.

As in our study many patients were treated with tricyclics and combination therapies, we did not find differences in therapy outcomes between aMDD and naMDD patients. The majority of aMDD patients responded to the therapy and could not be classified as aMDD at the second time point. However, some patients still showed elevated levels of anxiety, even after 7 weeks of treatment and thus could still be categorized as aMDD patients. Patients with worse outcomes showed higher anxiety scores which was particularly observed in patients treated with venlafaxine. Based on this observation, it may be useful to identify aMDD at the time point of admission to adapt the initial therapy (e.g., to avoid noradrenergic drugs such as venlafaxine) and include an anxiety-focused psychotherapy. In addition, our results suggest that categorization for aMDD versus naMDD using the ASF may be highly advantageous in clinical practice, particularly for severe cases of MDD patients without remission after a 7-week standard therapy, in order to augment the treatment with anxiety symptom-targeted methods.

The major limitation of our study is the naturalistic study design. This meant that the medications could not be analysed individually, as for many of the MDD patients more than one psychopharmacological medication was administered. Secondly, the sample size is relatively small, especially regarding specific drugs subgroups. There may only be small effect sizes for individual drugs, which can only be detected with a larger sample size. Thirdly, it is important to acknowledge that the treatment effects observed in our study may not be solely attributed to the antidepressant drugs administered, as some patients received parallel psychotherapy. Finally, the onset of therapy effects may be delayed beyond 7 weeks which may also have contributed to non-significant findings.

## 4. Materials and Methods

### 4.1. Patients

The GEParD (Genetics and Epigenetics of Pharmaco- and Psychotherapy in acute and recurrent Depression) sample consisted of 346 depressive patients recruited at the Department of Psychiatry, Psychosomatics and Psychotherapy of the University Hospital of Wuerzburg, Germany [34]. All participants were Caucasian, between 18 and 80 years of age, and diagnosed for unipolar or bipolar depressive disorder according to the Diagnostic and Statistical Manual of Mental Disorders (DSM)-IV [35] criteria. Patients with severe neurological or general medical conditions were excluded. A demographic overview of the GEParD sample is given in Table 1. A drug-specific summary is given in Supplementary Table S1. More detailed information has been reported elsewhere [34].

The GEParD study was approved by the local ethics committee of the University Hospital of Wuerzburg (104/12, amended 128/15). All participants were informed in detail and gave written informed consent to the evaluation and publication of clinical data in accordance with the ethical principles of the Helsinki Declaration (WMA 2013).

### 4.2. Definition of Anxious Depression

Anxious depression was defined as major depression with a high proportion of anxiety symptoms and assessed by the HDRS-21 anxiety-somatization index according to Cleary and Guy, 1977 [36]. The anxiety-somatization factor (ASF) consists of six HDRS-21 items: psychological anxiety, somatic anxiety, gastrointestinal somatic symptoms, general somatic symptoms, hypochondria and insight. For categorical analyses patients with an ASF of at least 7 were classified as aMDD patients, and an ASF of below 7 as naMDD.

### 4.3. Definition of Treatment Response

Antidepressant treatment was carried out according to the attending physician’s choice (naturalistic setting) for up to 7 weeks. Severity of depressive symptoms and their change during psychopharmacotherapy were assessed weekly, using the Hamilton Depression Rating Scale (HDRS)-21 [37,38]. Treatment response was defined by a reduction in the HDRS-21 score of at least 50% at week 7 or the time point of discharge from the study [37,39]. To ensure an independent assessment of anxiety levels from treatment response in depressed patients according to remission indicated by the HDRS-21 sum score, six of the HDRS-21 items used to define anxious depression (see the following section) were excluded from assessing response to treatment.

### 4.4. Drug Doses and Serum Concentrations

Drug doses and serum concentrations of week 1 and week 7 (or the last week of study participation) were analysed separately. Serum concentrations for therapeutic drug monitoring (TDM) were determined and defined according to the AGNP-TDM expert group consensus guideline [39]. For amitriptyline + nortriptyline and venlafaxine + O-desmethylvenlafaxine, sum serum concentrations were used, as they represent the active moieties (AM) of these drugs. An overview of administered drugs is given in Supplementary Table S2.

### 4.5. Statistical Analyses

Statistics and figures were performed in R v4.1.1 [40]. Dimensional outliers (≥4 SD from mean) from drug doses, serum concentrations and the ASF-score were set as missing data. To examine the influence of the dimensional ASF-score on treatment response, drug doses or serum concentrations, linear regression models were carried out by adjusting for age and sex. Additionally, the analyses were carried out with a correction for the following administered comedications: tricyclic antidepressants (TCA), selective serotonin reuptake inhibitors (SSRI), serotonin-norepinephrine reuptake inhibitors (SNRI), norepinephrine and dopamine reuptake inhibitors (NDRI), noradrenergic and specific serotonergic antidepressants (NaSSA), antipsychotics and antiepileptics. Comparisons of categorically grouped aMDD and naMDD patients regarding treatment response, drug doses or serum concentrations were performed by logistic regression models adjusted for age and sex. For all analyses, the nominal significance level (*p* ≤ 0.05) was Bonferroni-adjusted for the total number of analyses (26×). Computation of the statistical power was performed with G*Power v3.1.9.2 [41].

## 5. Conclusions

We did not find that anxious depression is associated with poorer treatment response in our naturalistic study. However, our study included combination therapies such as atypical antipsychotics and psychotherapy, which may have acted as extraneous variables. Nevertheless, the group of aMDD patients that showed a persistent aMDD even after 7 weeks of therapy showed a worse pharmacotherapy response than naMDD patients. Therefore, it may be beneficial to perform an initial early screen for aMDD and to supplement the treatment plan with anxiety symptom-targeted methods. Clinical practice could benefit from the categorization into aMDD and naMDD, especially in severe cases of MDD without remission after 7 weeks. We also found that aMDD patients at week 7 treated with venlafaxine had a significantly worse outcome compared to naMDD patients, potentially suggesting that treatment of aMDD should use a less noradrenergic drug and integrate anxiety-focused psychotherapy. Due to the inconsistent results of previous studies and the complexity of MDD subtypes, further prospective randomized controlled trials with larger sample sizes for individual drugs are necessary. 

## Figures and Tables

**Table 1:** Demographic overview of the total GEParD sample. Detailed drug-specific demographics for amitriptyline, mirtazapine, sertraline and venlafaxine are summarized in the Supplementary Table S1.

	N	Mean ± SD (Range) %/%
Included patients	346	
Age [years]	346	45.58 ± 15.31 (18–80)
Male/Female	146/200	42.2/57.8
Duration of disorder [years]	339	13.62 ± 12.31 (0–60)
HDRS-21 Baseline	346	25.06 ± 7.48 (4–46)
HDRS-21 Out	340	10.61 ± 6.81 (0–38)
Responder/Nonresponder	220/109	66.9/33.1
HDRS anxiety-somatization factor Baseline	342	6.68 ± 2.92 (0–15)
HDRS anxiety-somatization factor Out	332	2.8 ± 2.4 (0–10)
aMDD/naMDD Baseline	183/159	53.5/46.5
aMDD/naMDD Out	31/304	9.3/90.7

N, number of patients; SD, standard deviation; HDRS, Hamilton Depression Rating Scale; aMDD, anxious depressed patients; naMDD, non-anxious depressed patients.

**Table 2:** Summary of results for week 1 and week 7. Significant results are written in bold, adjusted *p*-values are given in brackets.

Week 1					Week 7			
	Dimensional (ASF-Score)		Categorical (aMDD vs. naMDD)		Dimensional (ASF-Score)		Categorical (aMDD vs. naMDD)	
	Beta	*p* (Adjusted)	Beta	*p* (Adjusted)	Beta	*p* (Adjusted)	Beta	*p* (Adjusted)
Treatment response in anxious depression								
Complete sample	0.05	0.27	−0.04	0.87	−0.32	4.67 × 10^−8^ (1.21 × 10^−6^)	−2.96	6.78 × 10^−7^ (1.76 × 10^−5^)
Drug doses in anxious depression								
Amitriptyline	0.01	0.30 (1)	0.01	0.44 (1)	−0.02	0.07 (1)	−0.01	0.26 (1)
Mirtazapine	0.02	0.53 (1)	0.00	0.92 (1)	−0.01	0.79 (1)	−0.02	0.63 (1)
Sertraline	−0.02	0.19 (1)	−0.02	0.13 (1)	−0.01	0.31 (1)	−0.27	1.00 (1)
Venlafaxine	0.00	0.17 (1)	0.00	0.09 (1)	0.00	0.92 (1)	0.00	0.95 (1)
Serum concentrations in anxious depression								
Amitriptyline	0.01	0.41 (1)	0.00	0.60 (1)	0.01	0.31 (1)	0.01	0.51 (1)
Mirtazapine	−0.01	0.69 (1)	0.00	0.86 (1)	0.00	0.88 (1)	0.01	0.47 (1)
Sertraline	0.02	0.30 (1)	0.18	0.48 (1)	0.02	0.03 (0.72)	0.03	0.02 (0.62)
Venlafaxine	0.00	0.34 (1)	0.00	0.09 (1)	0.00	0.09 (1)	0.00	0.21 (1)
Drug-specific treatment response in anxious depression								
Amitriptyline	0.08	0.45 (1)	−0.26	0.66 (1)	−0.30	6.98 × 10^−3^ (0.18)	−1.99	0.03 (1)
Mirtazapine	0.05	0.49 (1)	0.31	0.53 (1)	−0.53	1.76 × 10^−4^ (4.58 × 10^−3^)	−20.86	0.99 (1)
Sertraline	0.03	0.87 (1)	0.64	0.55 (1)	−0.55	0.05 (1)	−21.12	1.00 (1)
Venlafaxine	0.03	0.72 (1)	−0.53	0.20 (1)	−0.40	7.98 × 10^−6^ (2.07 × 10^−4^)	−3.11	2.89 × 10^−4^ (7.51 × 10^−3^)

ASF, anxiety-somatization factor; aMDD, anxious depressed patients; naMDD, non-anxious depressed patients; *p*, *p*-value.

## Data Availability

The data presented in this study are available on request from the corresponding author. The data are not publicly available due to privacy restrictions.

## Supplementary Materials

The following supporting information can be downloaded at: https://www.mdpi.com/article/10.3390/ijms242417128/s1.
