# Supplementary material for: Effects of Anxious Depression on Antidepressant Treatment Response
Source: Int J Mol Sci. 2023 Dec 5;24(24):17128. doi: 10.3390/ijms242417128 (PMC10742776; doi:10.3390/ijms242417128)
Supplement: Supplementary file 1 [file ijms-24-17128-s001.zip › ijms-2721414-supplementary.pdf]

Table S1. Demographic overview of drug-specific subsamples. Detailed drug-specific demographics for amitriptyline, mirtazapine, sertraline and venlafaxine for week 1 and week 7.

Week 1 (Baseline)	N	Mean ± SD (range)	N	Mean ± SD (range)
	Amitriptyline		Mirtazapine	
**Included patients**	68		110	
**Age [years]**	68	47.50 ± 13.06 (18-73)	110	48.75 ± 16.61 (18-80)
**male/female**	34/34		46/64	
**Duration of disorder [years]**	66	12.41 ± 10.94 (0.3-40)	106	14.18 ± 13.62 (0.3-60)
**Dose [mg]**	68	74.26 ± 43.33 (25-225)	110	26.18 ± 12.54 (3-60)
**Serum concentration [ng/ml]**	45	66.49 ± 51.65 (0-236)	70	34.97 ± 20.32 (6-110)
**Normal dose range [mg]**		50-150		15-45
**Normal serum levels [ng/ml]**		80-200		30-80
**HDRS-21 Baseline**	68	24.27 ± 7.53 (7-39)	110	26.85 ± 7.52 (9-46)
**HDRS-21 Out**	67	12.42 ± 7.25 (2-33)	109	10.14 ± 6.60 (0-27)
**Responder/Nonresponder (Out)**	37/29		71/37	
**HDRS anxiety-somatization factor Baseline**	68	6.60 ± 2.91 (1-13)	110	7.27 ± 3.16 (0-15)
**HDRS-21 anxiety-somatization factor Out**	82	3.23 ± 2.43 (0-9)	108	2.99 ± 2.51 (0-10)
**aMDD/naMDD Baseline**	41/27		68/42	
**aMDD/naMDD Out**	9/57		12/96	
	Sertraline		Venlafaxine	
**Included patients**	33		154	
**Age [years]**	33	52.33 ± 14.93 (19-75)	154	43.60 ± 14.32 (18-80)
**male/female**	12/21		65/89	
**Duration of disorder [years]**	32	14.63 ± 13.81 (0.5-49)	150	14.14 ± 11.95 (0.4-48)
**Dose [mg]**	33	78.94 ± 41.52 (5-200)	154	162.25 ± 87.63 (37.5-375)
**Serum concentration [ng/ml]**	19	30.21 ± 18.42 (4-73)	108	93.81 ± 124.40 (0-616)
**Normal dose range [mg]**		50-200		75-225
**Normal serum levels [ng/ml]**		10-150		100-400
**HDRS-21 Baseline**	33	25 ± 7.56 (11-39)	154	24.39 ± 7.66 (7-46)
**HDRS-21 Out**	33	11.72 ± 7.29 (1-33)	150	9.97 ± 6.35 (0-27)
**Responder/Nonresponder (Out)**	18/15		101/45	
**HDRS-21 anxiety-somatization factor Baseline**	33	6.85 ± 2.85 (1-14)	151	6.40 ± 2.88 (0-14)
**HDRS-21 anxiety-somatization factor Out**	33	3.18 ± 2.49 (0-10)	148	2.45 ± 2.34 (0-10)
**aMDD/naMDD Baseline**	17/16		74/77	

**aMDD/naMDD Out**	4/29		9/139	

Week 7 (Out)	N	Mean ± SD (range)	N	Mean ± SD (range)
	Amitriptyline		Mirtazapine	
**Included patients**	84		98	
**Age [years]**	84	46.90 ± 13.49 (18-75)	98	48.81 ± 16.59 (18-80)
**male/female**	39/45		43/55	
**Duration of disorder [years]**	82	12.13 ± 12.83 (0.5-60)	95	14.20 ± 13.34 (0-60)
**Dose [mg]**	84	107.74 ± 44.09 (25-225)	98	35.10 ± 12.26 (7.5-60)
**Serum concentration [ng/ml]**	71	82.44 ± 32.52 (14-151)	72	42.60 ± 17.12 (0-94)
**Normal dose range [mg]**		50-150		15-45
**Normal serum levels [ng/ml]**		80-200		30-80
**HDRS-21 Baseline**	84	25.96 ± 7.47 (7-43)	98	26.29 ± 7.45 (9-46)
**HDRS-21 Out**	83	12.77 ± 7.04 (2-38)	97	9.39 ± 6.54 (0-27)
**Responder/Nonresponder**	47/37		72/26	
**HDRS-21 anxiety-somatization factor Baseline**	84	6.93 ± 2.61 (1-13)	98	7.19 ± 3.07 (1-14)
**HDRS-21 anxiety-somatization factor Out**	82	3.43 ± 2.43 (0-10)	97	2.96 ± 2.48 (0-10)
**aMDD/naMDD Baseline**	52/32		59/39	
**aMDD/naMDD Out**	11/72		10/87	
	Sertraline		Venlafaxine	
**Included patients**	35		**166**	
**Age [years]**	35	47.37 ± 16.17 (18-75)	166	43.62 ± 14.48 (18-76)
**male/female**	12/23		67/99	
**Duration of disorder [years]**	35	11.09 ± 9.93 (0.2-41)	162	13.75 ± 11.97 (0.4-49)
**Dose [mg]**	35	145.71 ± 63.66 (25-250)	166	258.89 ± 98.89 (37.5-525)
**Serum concentration [ng/ml]**	33	69.12 ± 55.49 (0-242)	152	159.39 ± 144.05 (7-589)
**Normal dose range**		50-200		75-225
**Normal serum levels [ng/ml]**		10-150		100-400
**HDRS-21 Baseline**	35	26.83 ± 7.40 (9-42)	166	24.54 ± 7.68 (7-46)
**HDRS-21 Out**	35	11.31 ± 6.41 (1-25)	163	10.23 ± 6.50 (0-27)
**Responder/Nonresponder**	20/15		106/60	
**HDRS-21 anxiety-somatization factor Baseline**	35	7.71 ± 2.99 (1-14)	163	6.31 ± 2.78 (1-14)
**HDRS-21 anxiety-somatization factor Out**	34	3.12 ± 2.40 (0-10)	161	2.58 ± 2.42 (0-10)
**aMDD/naMDD Baseline**	22/13		79/84	
**aMDD/naMDD Out**	4/31		13/148	

N, number of patients; SD, standard deviation; HDRS, Hamilton Depression Rating Scale; aMDD, anxious depressed patients; naMDD, non-anxious depressed patients.

Table S2. Overview of all administered psychiatric drugs.

Psychiatric Drug	N (Dose)	N (Concentration)	Psychiatric Drug	N (Dose)	N (Concentration)
**Antidepressants**			**Antipsychotics**		
Venlafaxine	166	152	Quetiapine	162	131
Mirtazapine	98	72	Aripiprazole	26	20
Amitriptyline	84	71	Risperidone	23	12
Sertraline	35	32	Olanzapine	19	17
Bupropion	29	16	Promethazine	3	3
Escitalopram	22	20	Melperone	3	2
Clomipramine	16	14	Pipamperone	3	2
Trazodon	15	12	Perazine	2	0
Doxepin	13	13	Ziprasidone	1	1
Duloxetine	13	9	Clozapine	1	0
Milnacipran	5	4	**Antiepileptics**		
Nortriptyline	5	4	Pregabaline	33	17
Trimipramine	4	3	Lamotrigine	14	12
Fluoxetine	1	1	Valproic Acid	13	10
Maprotiline	1	1	Gabapentine	6	3
			Levetiracetam	1	1
			Zonisamide	1	1
			Topiramate	1	0

N, number of dose and serum concentration data available for the evaluation.
